# Serum from a Patient with GAD65 Antibody-Associated Limbic Encephalitis Did Not Alter GABAergic Neurotransmission in Cultured Hippocampal Networks

**DOI:** 10.3389/fneur.2015.00189

**Published:** 2015-08-28

**Authors:** Nelly Stemmler, Karin Rohleder, Michael P. Malter, Guido Widman, Christian E. Elger, Heinz Beck, Rainer Surges

**Affiliations:** ^1^Department of Epileptology, University Hospital Bonn, Bonn, Germany; ^2^Center for Rare Diseases Bonn (ZSEB), University Hospital Bonn, Bonn, Germany

**Keywords:** GAD65 antibodies, limbic encephalitis, GABAergic neurotransmission, hippocampal cultures, GABA(A) currents

## Abstract

**Background:**

Glutamate decarboxylase is an intracellular enzyme converting glutamate into GABA. Antibodies (abs) to its isoform GAD65 were described in limbic encephalitis and other neurological conditions. The significance of GAD65 abs for epilepsy is unclear, but alterations of inhibitory GABAergic neurotransmission may be involved. Here, we investigated the effects of the serum of a female patient suffering from GAD65 ab-associated LE on GABA_A_ currents in cultured hippocampal networks.

**Methods:**

Spontaneous or evoked post-synaptic GABA_A_ currents were measured in cultured hippocampal neurons prepared from embryonic mice after 11–21 days *in vitro* using the patch-clamp technique in the whole-cell mode after incubation with serum of a healthy control or the LE-patient at a final concentration of 1% for 5–8 h.

**Results:**

Properties of miniature inhibitory post-synaptic currents were not different in cultures treated with control and LE-serum. Likewise, paired-pulse ratio of evoked GABA_A_ currents as a measure of release probability was not different in both conditions. Evoked GABA_A_ currents were significantly depressed during 10 Hz stimulation without significant differences between control and LE-serum treated cultures.

**Conclusion:**

In our experimental paradigms, serum of a patient with confirmed GAD65 ab-associated LE had no apparent effect on GABAergic neurotransmission in murine-cultured hippocampal networks. These results challenge the view that the presence of GAD65 abs invariably compromise inhibitory network function.

## Introduction

Limbic encephalitis (LE) is clinically characterized by new onset of temporal lobe seizures as well as disturbances of mood and memory function. An increasing number of different auto-antibodies (abs) are detected in the serum and cerebrospinal fluid (CSF) of affected people. These abs include classical onconeuronal abs, but also directly target neuronal structures in extracellular and intracellular compartments (1).

Neuronal abs associated with LE typically bind to structures which are involved in the process of synaptic transmission or modulation of neuronal excitability. Extracellular antigens at the cell surface comprise NMDA receptors, components of voltage-gated potassium channels (VGKCs) as well as GABA_A_ and GABA_B_ receptors, which are likely to alter neurotransmission or neuronal excitability (2–4). At least for abs against NMDA and GABA_A_ receptors, abnormally impaired neurotransmission was recently shown in *in vitro* experiments mainly by internalization of the receptors and subsequently reduced ionic conductances (5–7). Abs against VGKCs appear to enhance synaptic transmission (8).

In contrast to the aforementioned surface antigens, glutamate decarboxylase (GAD) is an intracellular enzyme that converts the excitatory neurotransmitter glutamate into the inhibitory neurotransmitter GABA. Two isoforms of GAD are expressed in the brain, a 67 kDa isoform (GAD67) and a 65 kDa isoform (GAD65) (9). Interestingly, defects in GAD65 activity were associated with recurrent seizures in GAD65 knock-out mice (10). Abs against GAD65 were detected in the serum or CSF of people with diabetes mellitus type I, LE and other neurological conditions, such as stiff person syndrome (SPS) or cerebellar ataxia (11, 12). In view of the molecular weight and dimensions of IgG abs and the intracellular localization of GAD65 in synaptic terminals, it remains to be determined whether GAD65 abs interfere with GABAergic neurotransmission in the brain, thereby possibly enhancing excitability of neuronal networks and contributing, e.g., to the generation of epileptic seizures. From a clinical point of view, this question may be relevant when selecting immunomodulatory treatment options. If GAD65 abs do not directly influence brain function, they may rather reflect an epiphenomenon of the underlying autoimmune process and ab-removal by plasma exchange may not be helpful.

Previous experimental studies suggest that GAD65 abs can indeed interfere with GABAergic signaling. First, GAD65 abs inhibit enzymatic GAD65 activity (13, 14). Second, GAD65 abs can cross the blood–brain barrier, reach the brain tissue and appear to be bound and taken up by hippocampal neurons (15–17). Third, serum or CSF from patients with SPS or progressive cerebellar ataxia and GAD65 abs led to a rapid and reversible pre-synaptic inhibition of GABA release in rat cerebellar slices within 10–15 min upon acute application (18–21). Furthermore, the serum of an epilepsy patient with GAD65 abs induced a twofold increase of network activity of hippocampal cultures within 2–3 min after application (22). Finally, passive transfer experiments in rats showed that intracerebellar, intraventricular, or intrathecal administration of IgG abs from patients with SPS or cerebellar ataxia and GAD65 abs induced motor dysfunction in rats (23, 24).

Taken together, previous studies support the notion that abs targeting the intracellular GAD65 enzyme possibly alter inhibitory neurotransmission in people with LE and thereby facilitate the generation of recurrent epileptic seizures. Here, we investigated the effects of the serum from a female patient suffering from GAD65 ab-associated LE on spontaneous and evoked GABAergic neurotransmission in cultured hippocampal neurons.

## Materials and Methods

### Detection of antibodies

Identification of GAD65 abs was performed by radioimmuno-precipitation assay (RIA) using ^125^I-GAD (normal values <1 U/ml, laboratory of Professor Angela Vincent, Weatherall Institute, Oxford, UK) or by indirect immunofluorescence test (IFT, normal values <1:10, EUROIMMUN Laboratory, Luebeck, Germany) and enzyme-linked immunosorbent assay (ELISA, normal values <10 IU/ml, EUROIMMUN). Presence of VGKC-abs was tested by RIA (normal values <100 pmol/l, laboratory of Professor Angela Vincent and EUROIMMUN) and NMDAR-abs were tested by specific IFT (laboratory of Professor Angela Vincent).

### Cell cultures

Primary neuronal cultures were prepared from fetal (E16–E19) mice brains (C57BL/6N). Embryo heads were dissected and placed in ice-cold dissection medium (HBSS and 20% fetal bovine serum). The meninges were cautiously removed and the cortex and hippocampus of every pup were isolated and separately collected. Trypsin was added to a final concentration of 0.05% and incubated for 10 min at 37°C. Subsequently, the enzyme solution was removed, the preparations were washed and the gently dissociated cells were plated on coverslips coated with poly-l-lysine. Hippocampal neurons were grown in Eagle’s Basal Medium supplemented with 2% B27, 1% fetal bovine serum, 0.45% glucose, and 0.2 mM l-glutamine at 37°C and 95% O_2_/5% CO_2_. After 11–21 days *in vitro*, neuronal cultures were incubated with patient serum (containing more than 2000 IU/ml GAD65 IgG abs according to ELISA; see also Section “Results”) or control serum (a healthy 23-year-old woman) at a final concentration of 1% for 5–8 h prior to patch-clamp recordings. Experiments were performed for each condition in a blinded fashion (experiments and analysis of the raw data was performed blinded to the origin of the applied serum). Furthermore, experiments were matched for days *in vitro* and cell culture batch, i.e., both control and LE-serum were tested in cell cultures of the same batch and at the same days in order to avoid age- or maturation-dependent effects. All animal experiments were conducted in accordance with the guidelines of the Animal Care and Use Committee of the University of Bonn. The study was approved by the Local Ethics Committee and written informed consent was obtained from the patient.

### Electrophysiology

Cells were visualized using an inverse microscope (Axiovert 40 CFL, Zeiss, Jena, Germany). Somatic whole-cell voltage-clamp recordings were made with a HEKA EPC-9 patch-clamp amplifier (HEKA Electronics, Lambrecht, Germany). Electrode resistance in the bath varied between 3 and 4 MΩ. Series resistance was monitored throughout the experiment. Data were sampled at 10 kHz and filtered at 1 kHz. Holding potential was corrected on-line for liquid junction potentials.

To record miniature IPSC (mIPSCs), cells were held at −60 mV. Bath solution consisted of (in mM) 125 NaCl; 2.5 KCl; 2 CaCl_2_; 2 MgCl_2_; 30 glucose; and 25 4-(2-hydroxyethyl)-1-piperazineethanesulfonic acid (HEPES) (pH adjusted to 7.4, osmolality to 310 mOsmol). Overlapping excitatory ligand-gated currents and voltage-gated sodium channels were blocked by bath-application of 20 μM d(−)-2-amino-5-phosphonopentanoic acid (AP5), 10 μM 6-cyano-7-nitroquinoxaline-2,3-dione (CNQX) and 1 μM tetrodotoxin (TTX). Internal solution consisted of (mM) 135 KCl, 10 HEPES, 5 Na_2_-Phosphocreatine, 2 EGTA, 4 Mg-ATP, and 0.5 Na_2_-GTP (pH adjusted to 7.3 and osmolality to 290–300 mOsmol).

Evoked IPSC were stimulated with a bipolar electrode (Royem Scientific Limited, UK, FHC; CBAEC75 – concentric bipolar electrode) placed close to the recorded neuron (25). The position of the stimulation electrode was kept unchanged during individual experiments at a distance of about 300 μm to the post-synaptic neuron. In most experiments, stimulation currents were set to an amplitude of 1 mA and a duration of 1 ms at a frequency of 10 Hz using an external pulse generator (A-M Systems, Inc., Carlsborg, USA Isolated Pulse Stimulator, Model 2100). Bath solution was continuously oxygenated with 95% O_2_/5% CO_2_ and consisted of (mM) 125 NaCl, 3.5 KCl, 2 CaCl_2_, 1 MgCl_2_, 15 Glucose, 1.25 NaH_2_PO_4_, and 26 NaHCO_3_ (pH adjusted to 7.4, osmolality 310 mOsmol). To block overlapping excitatory currents, bath solution contained 20 μM AP5 and 10 μM CNQX. Internal solution consisted of (mM) 130 KCl; 5 Na_2_-Phosphocreatine; 10 HEPES; 2 EGTA; 4 Mg-ATP; 0.5 Na_2_GTP; and 5 lidocaine N-ethyl-chloride to block voltage-gated sodium currents (pH adjusted to 7.3 and osmolality to 290–300 mOsmol). Holding potential was set to −60 mV.

### Data analysis

Miniature IPSCs were automatically detected using an algorithm, which measured the peak amplitude and rise and decay times. Undetected events and false positives were corrected by visual inspection. Amplitudes of evoked IPSC were determined from baseline to the maximal downward deflection. Statistics were done using Mann–Whitney tests. Data are given as mean ± SEM.

## Results

### Clinical case

**Figure 1:**
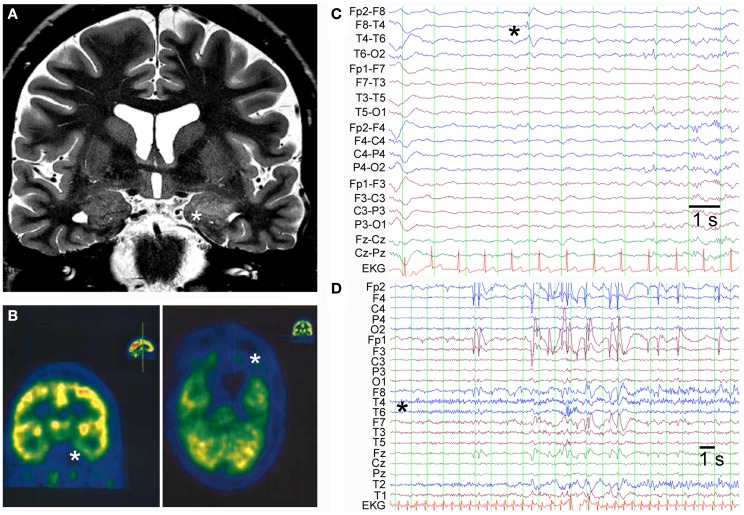
**(A)** Coronal T2-weighted cerebral MRI shows increased volume and signal in the left amygdala and anterior part of the hippocampus (indicated by asterisk). **(B)** FDG-PET displays hypometabolism in both temporo-mesial regions with a predominant deficit in the left hemisphere (indicated by asterisks: left panel coronal section and right panel axial section). **(C)** Interictal scalp EEG in bipolar montage shows sharp-slow-wave activity in the right fronto-temporal region (indicated by asterisk). **(D)** Ictal scalp EEG of a typical temporal lobe seizure demonstrates seizure-onset over the right temporal region with a regional maximum at the electrodes T6, T4, and T2 (unipolar montage against common average).

A 45-year-old female patient presented with temporal lobe seizures since the age of 43 years, a moderate depression as well as predominant deficits in visual memory pointing toward disturbed function of the right temporal lobe. Cerebral magnetic resonance imaging (MRI) at the time of disease-onset revealed increased volume and signal intensity in both temporo-mesial structures (amygdala and hippocampus), whereas 2 years later, MRI abnormalities persisted in the left mesial temporal lobe only with apparent regression on the right hemisphere (Figure 1A). In line with the MRI findings, cerebral 18-fluorodeoxyglucose-positron emission tomography (FDG-PET) showed decreased hypometabolism in both mesial temporal lobes with a more pronounced deficit in the left temporal lobe (Figure 1B). Interictal scalp EEG displayed intermittent sharp-slow-wave complexes over the right fronto-temporal region during sleep (Figure 1C). Furthermore, a typical temporal lobe seizure was recorded from wakefulness with onset over the right temporal region (Figure 1D). In complementary analyses, GAD65 abs were detected in both serum (titer >1000 U/ml; later on >2000 IU/ml) and CSF (9.2 U/ml) along with oligoclonal bands in the CSF. Abs against NMDA receptors or VGKCs were not found. An extended search for tumors remained negative. The final diagnosis of non-paraneoplastic GAD65 ab-associated LE was made and immunomodulatory therapy over a time period of 2 years, including plasma exchange as well as administration of cyclophosphamide, cortisone, and mycophenolate mofetil, was started. In the further course, epileptic seizures proved to be difficult to treat (anticonvulsant drugs used until the last follow-up 5 years after onset of LE included lacosamide, lamotrigine, levetiracetam, oxcarbazepine, perampanel, pregabalin, topiramate, valproic acid, and zonisamide).

### Spontaneous GABAergic currents in cultured hippocampal neurons

**Figure 2:**
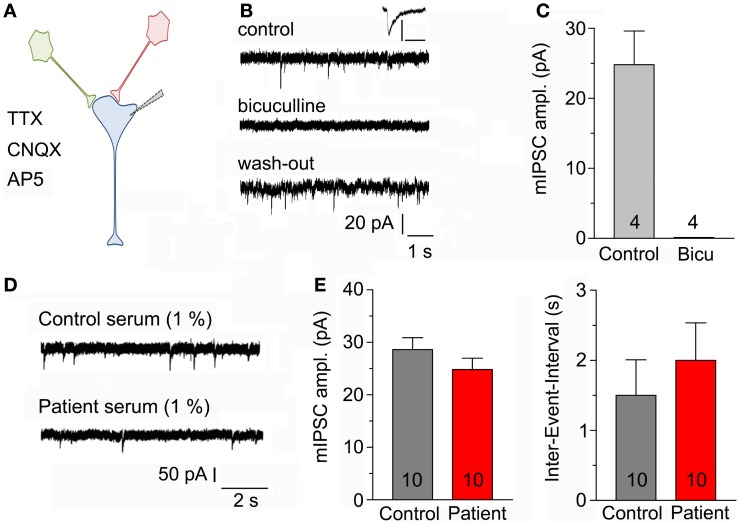
**(A)** Sketch of the experimental configuration. Miniature IPSCs are recorded with the help of a patch-clamp electrode in whole-cell mode in the presence of AP5, CNQX, and TTX in the bath solution (post-synaptic neuron in blue). **(B)** Original mIPSC traces before (upper panel), during (middle panel), and after application (lower panel) of 20 μM bicuculline to block GABA_A_ receptors. Inset displays enlarged control mIPSC, scaling 100 pA/100 ms. **(C)** Bicuculline completely blocked recorded post-synaptic currents (four recordings). **(D)** Original mIPSC-traces after incubation with control serum (upper panel) and patient serum (lower panel) both at a final concentration of 1%. **(E)** Amplitudes of mIPSC (left panel) and inter-event intervals (right panel) were not different after incubation with control and patient serum (10 recordings each condition).

Recordings were made in primary neuronal cultures after 11–21 days *in vitro* (Figure 2A). Spontaneous mIPSC were recorded during a time period of 5 min. Application of 20 μM bicuculline completely and reversibly blocked mIPSC (Figures 2B,C). To examine the effects of the serum of the above mentioned LE-patient (containing a GAD65 ab titer above 2000 IU/ml) on mIPSC, neuronal cultures were incubated with control or LE-serum at a final concentration of 1% for 5–8 h prior to recordings. Neither amplitudes of mIPSC (*p* = 1.0; control 28.5 ± 2.25 pA; LE-serum 25.9 ± 2.25 pA; 10 recordings each condition) nor inter-event intervals (*p* = 0.27; control 1.53 ± 0.49 s, LE-serum 1.98 ± 0.50 s; 10 recordings each condition) were different in both conditions (Figures 2D,E).

### Evoked GABAergic currents in cultured hippocampal neurons

**Figure 3:**
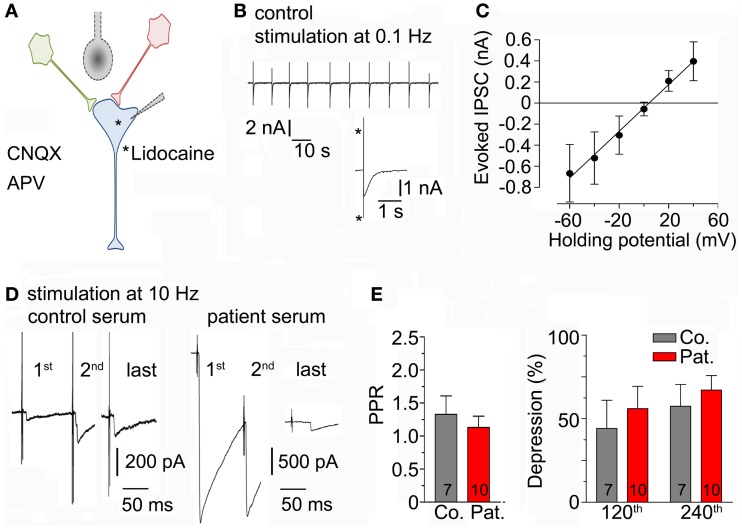
**(A)** Sketch of the experimental configuration. Evoked IPSCs were stimulated by an extracellular concentric bipolar electrode placed nearby the axons of surrounding neurons (in red and green) in the presence of AP5 and CNQX in the bath solution. The post-synaptic neuron (in blue) contained lidocaine to block voltage-gated sodium channels. **(B)** Original traces of evoked IPSC during extracellular repetitive stimulation at 0.1 Hz. Inset shows an enlarged evoked IPSC (asterisk indicate stimulation artifacts). **(C)** Amplitudes of evoked IPSC were measured at different holding potentials to determine the reversal potential (four recordings). **(D)** Original traces of evoked IPSC-traces during repetitive extracellular stimulation at 10 Hz for 2 min after incubation with control serum (left traces) and patient serum (right traces) at different time points. This figure illustrates the high variability between IPSC recordings from different cells (see also Section “Discussion”). **(E)** Paired-pulse ratio (PPR, ratio of second to first pulse; left panel) and post-synaptic depression at different time points (after 120 and 240 pulses, right panel) upon repetitive 10-Hz stimulation for 2 min were not different after incubation with control (7 recordings) and patient serum (10 recordings).

To test whether LE-serum affects GABAergic neurotransmission upon repetitive neuronal activity, a method to evoke synaptic release in neuronal cell cultures was established [see Section “Materials and Methods” and Figures 3A,B (25)]. To ascertain that GABAergic currents were measured in the experimental configuration, the calculated reversal potential for chloride (−0.09 mV) was compared to the experimentally determined reversal potential of the recorded post-synaptic currents (+4 mV, Figure 3C). Then, neuronal cultures were incubated for 5–8 h with control and LE-serum and GABAergic synaptic terminals were challenged by repetitive extracellular stimulation at 10 Hz for 2 min in both conditions (Figure 3D). Paired-pulse ratio (PPR, ratio of second to first pulse) as a measure of pre-synaptic release probability did not differ between control and LE-serum (*p* = 0.74; control 1.33 ± 0.28, 7 recordings; LE-serum 1.13 ± 0.17, 10 recordings). Evoked GABA_A_ currents were depressed during 10 Hz stimulation without significant differences between control and LE-serum treated cultures (synaptic depression at pulse 120 with respect to the first pulse: control 44.2 ± 16.8%, LE-serum 56.0 ± 13.4%, *p* = 0.47; at pulse 240: control 57.4 ± 13.0% LE-serum 67.1 ± 8.7%, *p* = 0.31; control 7 recordings, LE-serum 10 recordings; Figure 3E).

## Discussion

Previous studies in GAD65 knock-out mice suggested that the two isoforms of the GAD-enzyme, GAD65, and GAD67, have distinct roles in GABAergic neurotransmission: whereas GAD67 provides GABA for basal neurotransmission, GAD65 appears to be predominantly involved in the GABA synthesis upon sustained neuronal activity (26). In light of these findings, the lack of effects on mIPSC by the LE-serum during sparse, spontaneous activity may not be surprising. However, GABAergic transmission evoked with trains of electrical stimulation was also not affected by the LE-serum.

Our findings are at odds with the results of previous experimental studies which demonstrated a clear inhibition of GABAergic neurotransmission by serum, CSF or purified IgG in rat cerebellar slices (18–21). The lack of unequivocal alterations of GABAergic currents in our study could be due to the apparently high variability of the recorded post-synaptic currents and the relatively small sample size. In contrast to experiments using anatomically more intact neuronal preparations, such as hippocampal slices, neuronal cultures display a considerable variability (with respect to cellular composition, number of synaptic connections per neuron, etc.) and do not allow the unequivocal selection and identification of the same cell types. Thus, post-synaptic currents considerably varied from recording to recording in our experiments, as illustrated in Figure 3D, and may have masked a more subtle effect of GAD65 abs on GABAergic neurotransmission. The absence of clear-cut effects in our study is unlikely to be simply due to issues related to the concentration and duration of application. In our experiments, we have diluted the serum to a final concentration of 1%, which is similar to that in other studies (e.g., 18, 20). Higher concentrations of the patient’s serum (e.g., 2 or 10%) could have had measurable effects on GABAergic neurotransmission, but we have not tested this in our experiments. Regarding the duration of application, our neuronal cultures were pre-incubated for 5–8 h with the control and patient serum. Neuronal uptake of IgG abs was previously shown in cultured hippocampal neurons with a maximum after 4 h (15), well within our incubation times. It is theoretically possible that the human autoantibodies were not active in our study due to weak cross-reactivity between species. However, this has not been an issue in other cross-species studies that have claimed activity of patient serum or patient-derived abs in rodent preparations (18–21). Nevertheless, due to the lack of clear-cut effects on GABAergic neurotransmission, we have not performed further experiments to elucidate possible causes (i.e., we have not performed complementary immunohistochemical stainings to demonstrate failed neuronal uptake of abs or additional biochemical studies in order to investigate serum effects on enzyme activity of GAD65). One potential experimental confounder is the use of whole blood serum instead of purified abs. The patient serum contained the anticonvulsant drug levetiracetam at a concentration of about 150 μM, which was diluted to a final concentration of 1.5 μM. Levetiracetam binds to the synaptic vesicle protein 2A (SV2A) and presumably inhibits pre-synaptic calcium channels (27). In addition, run-down of GABA_A_ currents upon repetitive stimulation was attenuated in a *Xenopus oocyte* preparation by levetiracetam at low concentrations (28). The efficacious concentrations of levetiracetam, however, typically range from 35 to 100 μM (27), which makes it very unlikely that levetiracetam has exerted a measurable effect in our experiments.

Despite the limitations of the present study, our results suggest that GAD65 abs do not invariably modulate GABAergic neurotransmission in *all* patients that display these autoantibodies. This finding is supported by a recent experimental study, demonstrating that sustained GABAergic transmission and pre-synaptic GABAergic vesicle pool size remained unchanged upon application of IgG abs from SPS-patients with GAD65 abs (29). In other patients, however, clonally unrelated GAD65 abs may well interfere with GABAergic neurotransmission. LE associated with GAD65 abs may be heterogeneous with respect to the functional impact of GAD65 abs. This also suggests that it may be important to search for other inflammatory pathways, perhaps involving cellular or innate immunity that may play a role in the pathophysiology of altered hippocampal excitability in GAD65 ab-associated LE. In addition, regardless of whether GAD65 abs are functionally relevant as inhibitors of the GABA system, the diagnostic use as a biomarker for LE is still highly relevant, as it helps to make an early and correct diagnosis. Furthermore, the type of ab appears to have prognostic value and to predict the clinical course of the disease (30).

## Conflict of Interest Statement

The authors declare that the research was conducted in the absence of any commercial or financial relationships that could be construed as a potential conflict of interest.
